# Weight Loss Success With Repeat Intragastric Balloon Placement After Hyperinflation and Removal of the Index Balloon

**DOI:** 10.14309/crj.0000000000001071

**Published:** 2023-06-05

**Authors:** Michael A. Craig, Carl L. Kay, Kendra T. Stilwell, John G. Quiles

**Affiliations:** 1 Department of Internal Medicine, Brooke Army Medical Center, San Antonio, TX; 2 Department of Gastroenterology, Brooke Army Medical Center, San Antonio, TX

**Keywords:** adverse event, bariatric surgery, obesity, advanced endoscopy

## Abstract

Intragastric balloons are an increasingly common endoscopic alternative to bariatric surgery for the treatment of obesity. Hyperinflation is a rare complication that presents as acute-onset epigastric pain, nausea, vomiting, early satiety, abdominal distention or bloating, and rapid weight loss. Hyperinflation requires prompt diagnosis and removal of the balloon to prevent complications including gastric outlet obstruction or gastric perforation. We present a case of intragastric balloon hyperinflation with removal of the index device, followed by replacement with a second balloon, resulting in continued weight loss without further adverse events.

## INTRODUCTION

Approximately 40% of adults in the United States struggle with obesity, as defined by a body mass index (BMI) of ≥30 kg/m^2^. In the past decade, endoscopic bariatric surgery has become a valuable adjuvant treatment for weight loss, when combined with lifestyle modification.^1^ The American Gastroenterology Association guidelines of 2021 conditionally recommend intragastric balloon (IGB) therapy in addition to lifestyle modification over lifestyle modification alone in adults with a BMI between 30 and 40 kg/m^2^.^2^ However, IGBs are not without risks, such as perforation, gastric ulcers, gastric outlet obstruction, acute pancreatitis, and hyperinflation.^3^ Few cases of hyperinflation have been reported in the literature, but the cause and predisposing factors remain unclear.^4–6^ We present a case in which the patient's postprocedure course was complicated by hyperinflation requiring removal, who then elected to undergo repeat balloon placement. She had continued weight loss success without new complications.

## CASE REPORT

A 49-year-old woman with obesity (BMI of 35.5 kg/m^2^, 94 kg), dyslipidemia, prediabetes (hemoglobin A1c 5.8%), and hypertension (148/84 before antihypertensive medication) presented for evaluation of endoscopic bariatric treatment. The patient actively tried lifestyle and dietary modifications for 5 years without sustainable weight loss. After shared decision-making and discussion of risks and benefits, the patient elected to pursue endoscopic placement of an IGB for adjunctive antiobesity therapy. An Orbera IGB (Apollo Endosurgery, Austin, TX) was inserted through a standard endoscope, filled with 550 cc of sterile saline without air, and released into the stomach. Other than the presence of multiple small gastric polyps consistent with fundic gland polyps, no abnormalities were noted during endoscopy. The patient was started on pantoprazole 40 mg daily for mucosal cytoprotection from pressure ulceration and advised to avoid nonsteroidal anti-inflammatory drugs. She was scheduled for routine follow-up visits and repeat endoscopy in 6 months for IGB removal at the end of the standard treatment course.

The patient tolerated the balloon well for 4 months, losing 15 kg (79 kg, BMI 27.5 kg/m^2^). However, several days later, she presented to the emergency department with 48 hours of progressive nausea and vomiting. She had burning abdominal pain and constipation, and denied dysphagia, odynophagia, hematemesis, hematochezia, or melena. She had taken pantoprazole as prescribed and avoided nonsteroidal anti-inflammatory drugs as instructed. At time of emergency department presentation, she weighed 73 kg (BMI of 25.7 kg/m^2^).

**Figure 1.**
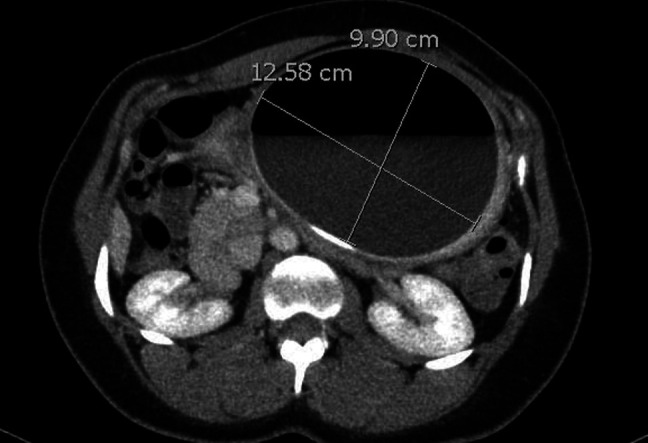
The hyperinflated intragastric balloon is vizualized within the stomach on computed tomography scan, measuring up to 12.58 cm in diameter.

**Figure 2.**
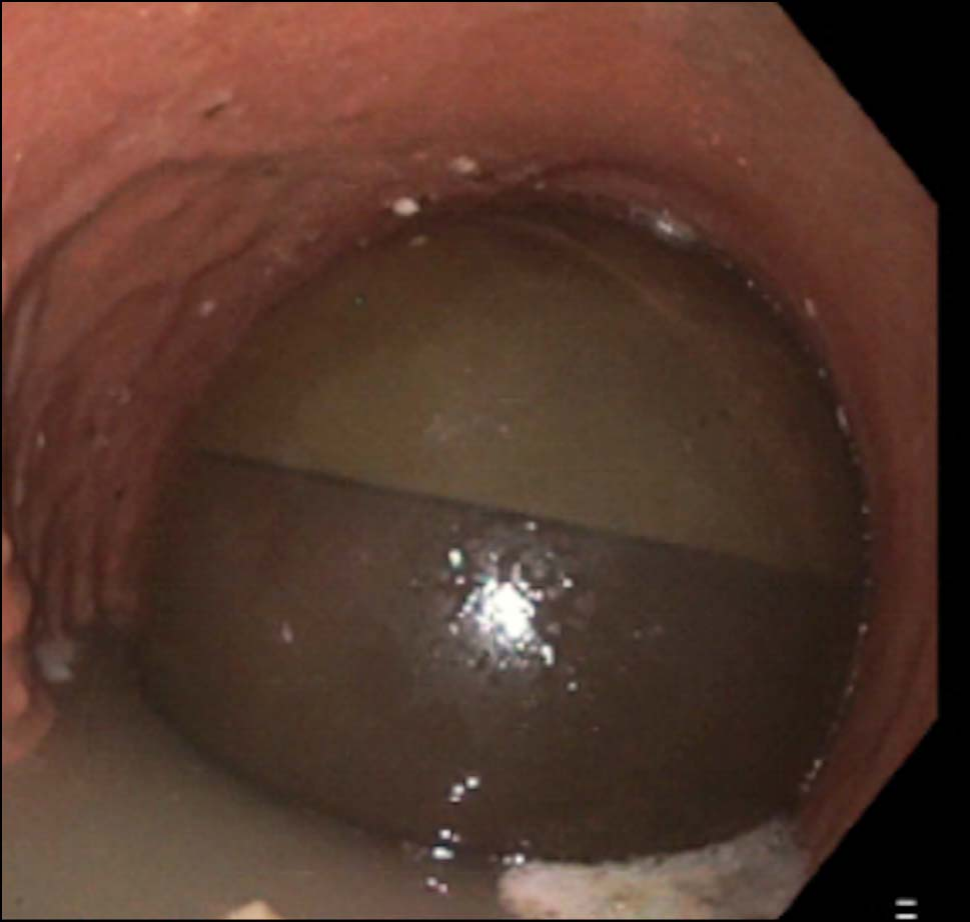
The hyperinflated intragastic balloon is seen within the stomach during endoscopy with prominent air-fluid level.

**Figure 3.**
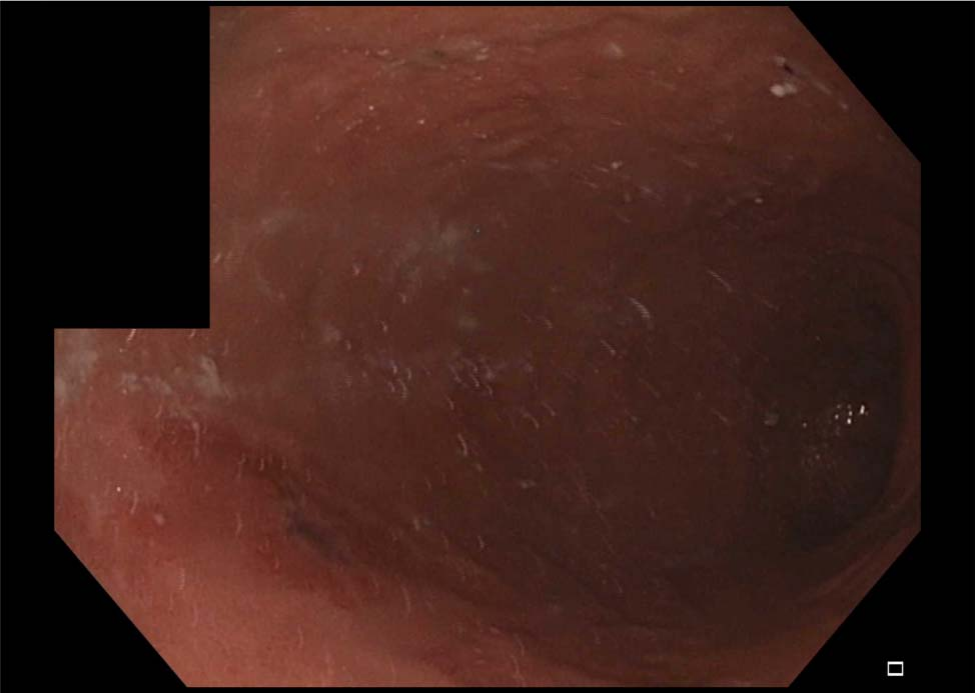
Gastric erosion after removal of hyperinflated initial gastric balloon.

On computed tomography, a large oval lucency was seen projecting over the left upper-quadrant abdomen measuring 12.6 × 9.9 cm, suggestive of hyperinflation of the IGB (Figure 1). Repeat upper endoscopy revealed an intact balloon in the body of the stomach, with an obvious air-fluid level within the balloon consistent with hyperinflation (Figure 2). The gastroscope was unable to traverse the IGB and enter the distal stomach or small bowel. After careful inspection, preparations were made for balloon removal. A suitable location was identified, and the Apollo aspiration needle (Apollo Endosurgery) was used to puncture the IGB. The stylet was removed, and the device was attached to suction until the balloon was completely deflated. Once deflation was complete, the balloon was captured with the Apollo Viper extraction device (Apollo Endosurgery) and removed through the mouth. Postinspection endoscopy demonstrated erythema with ulcers and erosions consistent with several 5- to 7-mm clean base ulcers without high-risk stigmata because of pressure from the balloon (Figure 3). Cultures of the IGB fluid were not obtained.

**Figure 4.**
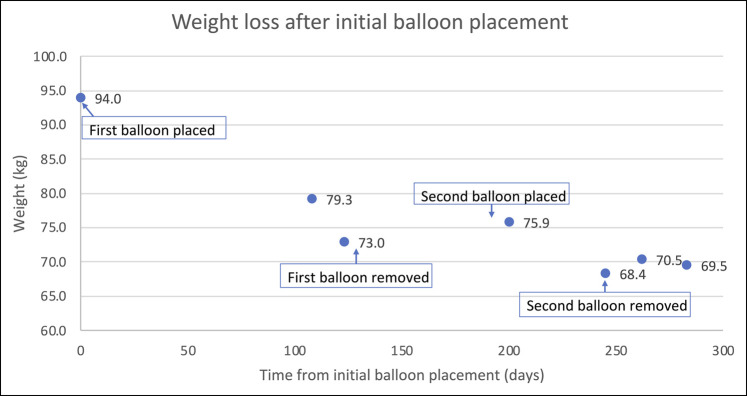
Weightloss over time with delineated events highlighted demonstrating weight loss with second initial balloon placement.

As she had only completed 4 of the planned 6 months, she requested to complete the remaining 2 months with a separate IGB. After allowing for mucosal healing of the pressure ulcers for 8 weeks, a second Orbera IGB was filled with 550 cc of sterile saline and remained in place 2 months to complete 6 total months. No complications occurred with the second IGB. It was removed after 6 total months and an additional 5.5-kg weight loss (68.4 kg, BMI 25.8 kg/m^2^) (Figure 4).

Fourteen months after placement of the initial balloon and 6 months after removal of the second, she has maintained weight loss, most recently weighing 69.5 kg (BMI 26.2 kg/m^2^). She has experienced tremendous improvement in comorbidities. Her blood pressure (126/74 off-antihypertensive therapy) and lipid panel have normalized off-medical therapy. Her most recent hemoglobin A1c was 5.2%, no longer prediabetic. Overall, she accomplished a 24-kg weight loss without the use of obesity medication. She reported smaller volumes of food, but her diet was not significantly altered. She felt enabled to exercise with increasing frequency after losing weight and stated she was delighted with her decision to pursue the second balloon placement.

## DISCUSSION

Hyperinflation is a rare but known complication of IGB placement, which presents as acute-onset nausea, vomiting, and epigastric abdominal pain typically outside the initial postprocedural window.^2^ The incidence of hyperinflation is unclear, but may occur in as many as 2.3% of patients.^3^ The postulated underlying pathophysiology likely stems from balloon and environmental factors. Balloon factors include permeability and fluid contamination.^5,7^ Environmental factors include gas-producing anaerobic bacteria and concurrent proton-pump inhibitor use.^5,8^ There is conflicting literature regarding the complex, synergistic role these factors may have in the development of spontaneous hyperinflation.^5–8^

We present a typical case of hyperinflation of an IGB, complicated by pressure ulcers, after which the patient elected to repeat placement of a second balloon with good metabolic response. This is the first documented case of successful replacement of an IGB after hyperinflation and removal of the first. It is unclear if hyperinflation is preventable. However, this case demonstrates a second attempt can be made without the same complication. Further studies are required before standardizing the decision to pursue a second balloon placement after hyperinflation.

Further studies are also needed to determine the underlying etiology of hyperinflation. Bacterial and fungal cultures of the IGB fluid should be considered because there are conflicting studies regarding microbial overgrowth as the cause of this complication.^7,8^ In the absence of recurrent hyperinflation, our case would argue against patient-related infection as a single causative factor. Therefore, we do not suggest any additional fungal or bacterial screening assessment if preplacement upper endoscopy seems endoscopically normal. There may be a future role for microbial screening (including *Helicobacter pylori* testing) if further literature solidifies a causative role. As IGB procedures for weight loss become more common, practitioners must be aware of this potential complication and options for further management.

## DISCLOSURES

Author contributions: MA Craig: analysis and interpretation of data, manuscript drafting, and is the article guarantor. CL Kay: acquisition of data and critical revision. KT Stilwell: acquisition of data and critical revision. JG Quiles: conception and critical revision.

Financial disclosure: None to report.

Other disclosure: The views expressed are those of the authors and do not reflect the official policy or position of Brooke Army Medical Center, the U.S. Army Medical Department, the U.S. Army Office of the Surgeon General, the Department of the Army, the Department of the Air Force, the Department of Defense, or the U.S. Government.

Previous presentation: Tri-Service American College of Physicians Annual Meeting; September 7, 2022; San Antonio, TX.

Informed consent was obtained for this case report.
